# A time series analysis of the relationship between ambient temperature and ischaemic stroke in the Ljubljana area: immediate, delayed and cumulative effects

**DOI:** 10.1186/s12883-021-02044-8

**Published:** 2021-01-14

**Authors:** Mirjam Ravljen, Fajko Bajrović, Damjan Vavpotič

**Affiliations:** 1 University of Ljubljana, Faculty of Health Sciences, Zdravstvena pot 5, Ljubljana, Slovenia; 2 University Medical Centre Ljubljana, Neurology Clinic, Department of Vascular Neurology and Neurological Intensive Care, Zaloška cesta 2, Ljubljana, Slovenia; 3 University of Ljubljana, Faculty of Computer and Information Science, Information Systems Laboratory, Večna pot 113, Ljubljana, Slovenia

**Keywords:** Stroke, Weather, Morbidity, Central Europe, Average daily ambient temperature, Time lag

## Abstract

**Background:**

Stroke is a major health problem around the world. Several studies have examine the influence of ambient temperature on incidence of stoke, but they reported different results for different types of stroke and different geographical regions. Hence, effect of ambient temperature is still much of interest, when focusing on ischemic stroke (IS) in regions that have not been examined yet. The aim of our study is to analyse association between IS incidences and short, delayed and cumulative effect of average daily ambient temperature, humidity and pressure in central Europe. To the best of our knowledge, this is the first IS study conducted between 45° and 50° latitude where large part of Central European population resides.

**Methods:**

We linked daily hospitals’ admission data for whole population and separately for two specific age groups with ambient temperature data. We considered patients coming from Ljubljana basin and its immediate surrounding. Data were gathered daily from January 2012 to December 2017. To measure the effect of average ambient temperature, humidity and pressure we used generalized linear model with a log-link-function and a Poisson distribution.

**Results:**

The results of our study show a statistically significant immediate, delayed and cumulative effects of ambient temperatures on IS incidence for the whole population and the population older than 65 years. Specifically, 1 °C reduction in ambient temperature on a given day (Lag 0) increases the IS risk for approximately 5‰ (all population) or 6‰ (population older than 65 years). Similar effects were found for lags from 1 to 6. Analysis of time windows from 0 to 1 days up to 0–28 days also show statistically significant cumulative effect for the same two age groups. IS incidence was not found to be significantly related to pressure or humidity in any group.

**Conclusion:**

The findings of this study may help healthcare authorities in central Europe improve existing stroke prevention measures and raise public awareness.

## Background

It has been known for many decades that low and/or high ambient temperature influences the risk of morbidity and mortality worldwide. Especially the influence on the acute myocardial infarction incidence has been well examined [1] while the impact of ambient temperature on stroke morbidity appears to have received less attention. The systematic review made by [2] from inception through to the year 2015 indicates the first study was made by [3], who started to collect data in the year 1961. Since then, several studies have been conducted in different geographical regions and reported inconsistent results [4]. Remarkably, [5] in their systematic review and meta-analysis reported both hot and cold temperatures association. A recent meta-analysis [2] provides evidence that the geographic latitude is the most significant source of this inconsistency. However, their meta-analysis shows a lack of ischaemic stroke (IS) studies on population living in a belt between 45°N and 50°N latitude. This belt is of special interest for Central Europe as many capitals and large cities like Bratislava, Budapest, Munich, Stuttgart, Vienna, and Zurich etc. are located here.

Annually, 15 million people worldwide suffer a stroke [6]. It is caused either by a clot in an artery supplying blood to the brain resulting in ischemia or when a burst vessel causes blood to leak into any part of the brain leading to hemorrhage [7]. Based on that stroke can be classified as ischemic or hemorrhagic stroke (HS). Since the pathological background is different, the effects of meteorological factors on IS may also differ from the effects of meteorological factors on HS [8]. Therefore, future studies should examine IS and HS risk separately [9].

In line with the aforementioned findings, our study focuses on daily IS incidence in central Europe in the latitude belt between 45°N and 50°N. We examined the immediate, delayed and cumulative effects of ambient temperature, humidity and pressure concerning IS incidence.

## Methods

Köppen-Geiger climate classifications [10] shows that the majority of central Europe including most of Austria, Czech Republic, Germany, Hungary, Poland, Slovakia, Slovenia and Switzerland have a common humid continental climate. This type of climate has warm to hot summers and cold winters. Our study was conducted in Ljubljana, the capital of Slovenia located in central Europe at latitude 46°N.

Daily IS incidence data was collected from University Medical Centre Ljubljana, which is the leading healthcare institution in Slovenia. The patients came from Ljubljana area and its immediate surrounding where approx. 360,000 inhabitants live at altitude between 300 and 400 m above sea level. Geographically the observed area is a basin with relatively homogeneous weather conditions. The average daily ambient temperature, humidity and pressure data were gathered from the Slovenian Environment Agency meteorological station located approximately in the middle of the observed area.

We focused on the average daily ambient temperature which is suggested to be the best exposure measure as it represents the exposure throughout the whole day and can be easily interpreted [11, 12].

Many studies consider only ambient temperature [4, 13–17] which is seen as the best exposure measure [11, 12] however other environmental variables like air pressure and humidity are also often considered [18].

We observed 2943 IS patients between 1st of January 2012 and 31st December 2017 whose discharge diagnoses were coded from I63.0 to I63.8 according to the 10th revision of the International Classification of Diseases (ICD-10). Among them 2380 patients were older than 65 years and 563 younger than 65 years.

We followed well established approach based on time series analysis using a main effect generalized linear model to relate data on the incidence of IS with ambient temperature, humidity and pressure data. The used generalised linear model assumes a log-link function with the Poisson distribution which is appropriate for counts of observations (number of IS events per day).

All procedures performed in study were in accordance with the ethical standards of the national research committee (National Medical Ethics Committee, reference number: 0120–567/2018/5) and with the 1964 Helsinki declaration and its later amendments or comparable ethical standards.

## Results

The results of the analysis of the possible link between each of the three meteorological variables and the incidence of IS showed that only the association between ambient temperature and incidence of IS is significant while neither pressure nor humidity link to IS incidence was found to be significant.

Table 1 and Table 2 show the results linking average daily ambient temperatures with the daily IS incidence for the entire population and for the population under and over 65 years of age. For all three groups we considered up to 7-day lags and different time windows. Tests of over-dispersion confirmed the Poisson model assumptions and also showed a statistically significant goodness of fit and Omnibus test *p*-value below 0.05. Such results indicate a relevant improvement of the fitted model over the intercept-only model.

**Table 1:** Ischemic stroke incidence in relation to the daily ambient temperature from 0-day lag to 6-day lag for the entire population, population older than 65 years and population younger than 65 years

Parameter	All population				> 65 years^**2**^				≤ 65 years^**3**^			
	Β	Sig.	Exp (B)	Omnibus test	Β	Sig.	Exp (B)	Omnibus test	Β	Sig.	Exp (B)	Omnibus test
Lag 0	-.005	.017	.995	.018	-.006	.011	.994	.011	-.001	.851	.999	.851
Lag 1	-.006	.010	.994	.010	-.006	.011	.994	.011	-.001	.777	.999	.777
Lag 2	-.006	.011	.994	.011	-.007	.007	.993	.007	-.001	.830	.999	.830
Lag 3	-.005	.026	.995	.026	-.006	.013	.994	.013	-.000	.981	1.000	.981
Lag 4	-.004	.046	.996	.046	-.005	.038	.995	.038	-.002	.759	.998	.759
Lag 5	-.006	.011	.994	.011	-.006	.021	.994	.021	-.006	.278	.994	.278
Lag 6	-.004	.046	.996	.046	-.005	.055	.995	.055	-.003	.535	.997	.535
Lag 7	-.004	.053	.996	.053	-.005	.054	.995	.054	-.002	.998	.998	.636

**Table 2:** Ischemic stroke incidence related to the average daily ambient temperature for different time windows for the entire population, population older than 65 years and population younger than 65 years

Β	All population				> 65 years^**2**^				≤ 65 years^**3**^			
	Β	Sig.	Exp (B)	Omnibus test	Β	Sig.	Exp (B)	Omnibus test	Β	Sig.	Exp (B)	Omnibus test
Windows W01	-.006	.010	.994	.010	-.007	.005	.993	.005	-.001	.899	.999	.899
Windows W03	-.006	.008	.994	.008	-.008	.003	.992	.003	.000	.956	1.000	.956
Windows W07	-.006	.006	.994	.006	-.008	.002	.992	.002	.000	.948	1.000	.948
Windows W014	-.007	.005	.993	.005	-.008	.002	.992	.002	.000	.986	1.000	.986
Windows W021	-.007	.005	.993	.005	-.008	.002	.992	.002	-.001	.876	.999	.876
Windows W028	-.007	.005	.993	.005	-.008	.002	.992	.002	.000	.973	1.000	.973

Results presented in Table 1 show that in case of all population beta coefficients up to 6-day lag were significant, while for population older than 65 years only coefficients up to 5-day lag were significant. None of the beta coefficients were significant for the population younger than 65 years. We can assume that 1 °C reduction in ambient temperature on a given day (Lag 0) increases the IS incidence for approximately 5‰ for all population, while for population older than 65 years it increases the IS incidence for approximately 6‰. Results for other lags can be found in Table 1.

The results in Table 2 show the relation between cumulative average daily temperature and increase in IS incidence. There is significant correlation between the two for entire population and population older than 65 for all the observed windows.

## Discussion

Our results show a significant correlation between average daily temperatures and IS incidence. We detected an increase in IS risk by approximately 5‰ in the case of a 1 °C decrease in average daily ambient temperature for the whole population on the same day. Eight of the related studies [3, 8, 9, 15, 19–22] similarly to our study report a significant negative correlation between ambient temperature and IS incidence. However, five studies report a significant positive association between ambient temperatures and IS incidence [23–27] and there are also five studies that did not detect any significant effect [13, 28–31]. Interestingly, only one of the five studies reporting positive association was performed above the latitude 40°N while studies report negative association from latitudes above and below 40°N. Additionally, a review and meta-analysis of the related studies [5] showed that temperature changes acted as risk factors in the negative and positive direction for IS. These findings strongly indicate that both increase and decrease of ambient temperatures can play an important role in IS incidence. However, it seems that the direction of this association is at least partially dependent on the latitude [2] which is probably further related to high and low average ambient temperatures that are typically observed at certain latitudes.

Although both temperature increase and decrease can play an important role in IS incidence, different factors explaining these effects have to be considered. In the case of studies that detected negative association between ambient temperature and IS incidence several factors which are triggered by lower ambient temperatures were proposed to increase IS incidence. These include vasoconstriction of the peripheral blood vassals [4, 5, 32], increased systemic vascular resistance [4, 8], and blood pressure rise [12, 32]. Moreover, increased sympathetic nervous system activity [12] results in increased platelet counts [5, 8] and increased blood viscosity [2, 32], which were both found to increase the risk of IS. Additionally, several of thrombogenic factors may rise, such as blood cell counts, plasma cholesterol, C-reactive protein, and fibrinogen concentrations [33], and also several physiological parameters may change, such as serum lipid, blood glucose, and serum fibrinogen concentration [5].

However, in the case of studies that detected positive association between IS incidence and ambient temperature, other factors may a play more important role. For instance, [26, 27] report that exposure to high ambient temperature is likely to cause dehydration, increase the risk of hemoconcentration and hyperviscosity resulting in thromboembolism and IS.

Next, our study examined specific population groups based on age. A significant association between average daily ambient temperature and IS incidence was only detected for population older than 65 years while no significant effect was detected for those who were less than 65 years old. The results show that IS incidence for the population older than 65 years increases by approximately 6‰ in the case of a 1 °C increase in daily average ambient temperature. Several studies reported similar results for the population group of older adults, reporting a stronger influence of low average temperatures on the occurrence of IS. A systematic review and meta-analysis [4] found that three studies reported subgroup analysis by age [9, 26, 34] with a stronger association between low average ambient temperature and IS in older patients. Moreover, several studies found that older people are more sensitive to the change of temperature because of the slower reaction of the regulation system [5, 35, 36].

Additionally, our results show delayed negative effects of average ambient temperature on IS incidence. We observed delayed increase in IS risk between 4 to 6‰ in the case of a 1 °C decrease in average ambient temperature one to 6 days prior to IS occurrence for all population and increase in IS risk between 7 and 8‰ one to 5 days prior to IS occurrence for population older than 65 years. Only a few studies analyse the delayed effect of ambient temperature on IS incidence. However, they do not examine the delayed effect on population older than 65. While Luo et al., 2018 reported a significant delayed harmful effect of cold ambient temperature on IS at 7 day lag for the whole population, other lag studies [28, 37] did not detect any significant correlation between IS incidence and ambient temperature.

Our study also detected the effect of cumulative average ambient temperature decrease on IS incidence. We observed 6 to 7‰ increase in IS risk in different time windows (0- 1, 0- 3, 0- 6, 7- 14) for all population and 7 to 8‰ increase for population older than 65. Other studies also analysed the cumulative effect using different time windows. For instance, several studies [8, 9, 14, 38, 39] report that decreases in average ambient temperatures for different time windows are associated with higher admission rates.

The link between IS and ambient pressure or humidity did not prove significant. This is in line with a systematic review and meta-analysis [18] which shows that there is no evidence of a relationship between ambient pressure or humidity and the occurrence of hospital admission for IS.

## Conclusion

Results of our study showed a statistically significant immediate, delayed and cumulative effect of decreases of average ambient temperatures on occurrence of IS for all population and population older than 65 years. We can assume that decreases in ambient temperatures expose this patients to higher risk of IS. The findings of our study analysing immediate effects of lower ambient temperature on the risk of the onset of IS correspond to the findings of the majority of other studies, however to the best of our knowledge, none of the existing studies analysed a delayed and/or a cumulative effect in a central Europe at latitude belt between 45°N and 50°N. Based on the results of our study we can suggest that population in central Europe should be more aware of IS risks associated with the decrease of ambient temperature and particularly of its cumulative effect. Special consideration should be given to population older than 65 years.

It is important to note a limitation of our study related to ambient temperatures measured at meteorological station. These temperatures may only partially reflect actual exposure of patients as they could be affected by indoor microclimate or they could travel to other locations in the observed time periods. To at least partially address this issue, our study includes only patients with permanent residence in Ljubljana basin area with relatively homogeneous weather conditions. Another limitation is that we were not able to consider air pollution as representative data for the studied area was unavailable.

The findings of this study may help healthcare authorities in central Europe improve existing stroke prevention measures and raise public awareness. The research in this field should continue to monitor how climate changes especially changes of average temperatures affect IS occurrence.

## Data Availability

The datasets used and/or analysed during the current study are available from the corresponding author on reasonable request.

## Abbreviations

IS: Ischemic stroke
HS: Hemorrhagic stroke
